# Dietary diversity and associated factors among children 6-23 months of age in Gorche district, Southern Ethiopia: Cross-sectional study

**DOI:** 10.1186/s12887-016-0764-x

**Published:** 2017-01-09

**Authors:** Dalecha Dangura, Samson Gebremedhin

**Affiliations:** 1 Sidama Zone Health Department, Hawassa, Ethiopia; 2 Hawassa University, School of Public and Environmental Health, Hawassa, Ethiopia

**Keywords:** Dietary diversity, Infant and young child feeding, Nutrition education, Husband involvement

## Abstract

**Background:**

Dietary diversity (DD) is useful indicator of dietary quality and nutrient adequacy. In developing countries limited evidence is available regarding predictors of DD during the critical complementary feeding period. The purpose of the study is to assess DD and predictors among children 6–23 months of age in rural Gorche district, Southern Ethiopia.

**Method:**

A community based cross-sectional study was conducted among 417 children aged 6–23 months in Gorche district. The children were selected using a stratified two-stage cluster sampling technique. DD in the preceding day of the survey was assessed using the standard 7-food group score without imposing a minimum intake restriction. Factors associated with DD were identified by modeling dietary diversity score (DDS) using linear regression analysis.

**Results:**

Only 10.6% (95% CI: 7.6–13.6) of the children had the minimum recommended DD (≥4 food groups). In children born to literate fathers, the DD was increased by 0.26 as compared to their counterparts (*p = 0.026*). Children from households that grow vegetables and own livestock, the DDS was significantly increased by 0.32 (*p = 0.032*) and 0.51 (*p = 0.001*). As the age of the child increases by a month, the DD also increased by 0.04 (*p = 0.001*). Mothers that received Infant and Young Child Feeding (IYCF) education during their post-natal care, the DDS was increased by 0.21 (*p = 0.037*). Unit increase in maternal knowledge on IYCF was associated with 0.41 rise in DDS (*p = 0.001*). Other factors that showed positive association were: mother’s participation in cooking demonstration, exposure to IYCF information on the mass media and husband involvement in IYCF.

**Conclusion:**

Nutrition education, promotion of husbands’ involvement in IYCF and implementation of nutrition sensitive agriculture can significantly enhance DD of children.

**Electronic supplementary material:**

The online version of this article (doi:10.1186/s12887-016-0764-x) contains supplementary material, which is available to authorized users.

## Background

In 2013, globally an estimated 6.3 million children under the age of 5 years died, 2.9 million of them in the Sub-Saharan Africa (SSA) region. In 2030 about 60% of all child deaths will be expected to occur in the region [1]. Worldwide, nearly half (45%) of all child deaths are linked to malnutrition and the figure might even be higher in Africa [2]. In SSA underlying causes of malnutrition include, but not limited to, poverty, adverse climatic conditions, natural resource degradation, fragile and poorly accessible health care services and existence of socio-cultural misconceptions [3].

In Ethiopia, over the past 15 years notable decline in child under-nutrition had been witnessed. Stunting was reduced by more than 30% and underweight was slashed by 40% [4]. Yet, childhood malnutrition still remains a major public health challenge. As of 2014, 9, 25 and 40% of children under the age of 5 years were wasted, underweight and stunted, respectively [4]. National level studies indicated nearly half of preschool children (44%) [5] and more than one-third of children 6–71 months (38%) have anemia and vitamin A deficiency [6].

Complementary feeding is a critical period in which malnutrition starts to develop in many infants, contributing significantly to the high burden of malnutrition in pre-school children [7]. In many developing countries complementary feeding practices are frequently ill-timed, unsafe and lack the desirable amount, feeding frequency and nutrient density for optimal child growth and development [8]. Globally, ensuring optimal complementary feeding can avert a substantial proportion of childhood deaths [9].

Dietary diversity (DD), the number of food groups consumed over a reference period [10], is a useful indicator of dietary quality, nutrient adequacy and nutritional status of children [11–13]. In the context of infant and young child feeding (IYCF), minimum DD – proportion of children 6–23 months of age who received foods from four or more out of seven standard groups in the preceding day– is as an imperative indicator [14]. However, in many low income countries meeting minimum the DD standard has been a major challenge. Summary of many Demographic and Health Surveys (DHS) conducted in developing countries witnessed that only a quarter of children met the requirement [15]. The figure is as low as 5% in Ethiopia [5].

Accordingly, the purpose of the current study is to assess dietary diversity and associated factors among children aged 6–23 months in Gorche district, Southern Ethiopia.

## Methods

### Study setting

The study was conducted in Gorche, one of the districts of Sidama Zone, Southern Ethiopia. According to the 2007 national census, the district has a population size of 139,780 of which 98% dwell in rural areas [16]. The livelihood of the population is reliant on subsistent mixed farming and the area is vulnerable to food insecurity. Major crops grown in the district are enset (Enset ventricosum), barely and broad beans. Agro-ecologically, the district is divided into midlands (20%) and highlands (80%). Administratively, Gorche is organized in 22 kebeles. A kebele is the smallest administrative unit in Ethiopia with an approximate 1,000 households.

### Study design

A community based cross-sectional quantitative study with both descriptive and analytic elements was used.

### Sample Size

The study was designed to include 417 children 6–23 months of age. The sample size was estimated using single population proportion formula with the following specifications: 95% confidence level, 14.4% expected prevalence of acceptable DD [17], 5% margin of error, design effect of 2 and 10% contingency to account for possible non-response. The adequacy of the sample size for identifying correlates of DD was evaluated using Gpower software [18].

### Sampling procedure

The study followed stratified two-stage cluster sampling procedure. Initially the available 21 rural kebeles were stratified into two groups – midlands and highlands – based on their predominating agro-ecological characteristics. From the available four and seventeen kebeles from the two strata, two and six kebeles were selected respectively using simple random sampling technique. Then, in each of the selected kebele, exhaustive listing of eligible children was made and independent sampling frame was developed. Ultimately children were selected using systematic random sampling procedure. The sample size of the study was proportionally distributed to the kebeles according to their population size.

### Data collection

Data were collected from the mothers/caregivers of the selected children using structured questionnaire prepared in the local language. The English version of the questionnaire is attached as a supplementary file (Additional file 1). Socio-demographic questions were adopted from the standard DHS questionnaire [5]. Minimum acceptable DD was defined as taking four or more food groups in the preceding day of the survey out of the seven standard food groups recommended by the WHO [19] without imposing a minimum intake restriction. Dietary diversity score (DDS) was computed by summing the number of unique food groups the child received in the preceding day of the survey. Household food security was measured and classified as recommended by the Food and Nutrition Technical Assistance (FANTA) Guideline [20].

Husbands were considered to be involved in IYCF when they perform at least two of the following four activities: (1) buying/bringing nutritious foods (egg, milk and meat) to feed the child, (2) giving money to his wife to purchase nutritious foods specifically to the baby, (3) checking whether his child is getting adequate amount of food or not, and (4) discussing with his wife about the type of food that should be provided to the child. Mothers were considered to be knowledgeable on IYCF when they know at least two from following four parameters: timely initiation of complementary food, dietary diversity, duration of breast feeding and timely initiation of family food.

Household wealth status was measured based on composite variables including ownership of selected household assets, size of agricultural land, quantity of livestock, materials used for housing construction and ownership of improved toilet and water sources.

### Data analysis

The data were entered into Epi-info 2007 software and statistical analysis was performed using SPSS version 20. Household wealth index was computed using Principal Component Analysis. Factors associated with DD were identified by modeling DDS using multivariate linear regression model. The association between each independent and dependent variable was initially assessed in simple (i.e. bivariate) regression model; then, variables that showed significant association were considered for multivariate model. The independent variables were modeled in two – distal and proximal – regression models. All socio-demographic characteristics and variables related agricultural production and exposure to IYCF education were considered as distal variables; whereas, maternal knowledge and husband’s involvement in IYCF were taken as proximal factors. Assumptions of the regression model (linearity, absence of multicollinearity, normality and homoscedasticity of error terms) were checked as described elsewhere and they were found to be satisfied [21]. Model fitness assessed using adjusted r-squared value. Outputs of the analysis were provided in unstandardized regression coefficients.

### Ethical consideration

Ethical clearance was secured from the Institutional Review Board (IRB) of Hawassa University, College of Medicine and Health Sciences. Data were collected after taking informed consent from the primary caregivers of the study subjects.

## Results

### Socio-demographic characteristics

From 417 infants and young children included in the study, 52.3% were males. The mean (±SD) age of the children was 15.3 (±5.6) months. The mean (±SD) household size of the study participants was 5.3 (±1.8) persons. More than half (54.0%) of households had two or more under five children. The majority (98.6%) of the children were sampled from male-headed households. Nearly all (92.3%) of the mothers were housewives and 81.5% had no formal education (Table 1).

**Table 1:** Socio-demographic characteristics of the respondents and study children in Gorche district, Southern Ethiopia, 2015

Variables (*n* = 417)	Frequency	Percentage
Head of the household		
Male	411	98.6
Female	6	1.4
Mother’s age (year)		
Below 25	102	24.5
25 and above	315	75.5
Child’s age (months)		
6–8	58	13.9
9–11	61	14.6
12–23	298	71.5
Child sex		
Male	218	52.3
Female	199	47.7
Father’s education		
Illiterate	294	70.5
Literate	117	28.1
Mother’s education		
Illiterate	340	81.5
Literate	77	18.5
Father’s occupation		
Farmer	398	95.4
Merchant	93	22.3
Others	9	2.2
Mother’s occupation		
Farmer/house wife	385	92.3
Merchant	55	13.2
Others	3	0.7
Marital status		
Married/living together	411	98.6
Widowed	6	1.4
Family size		
Less than five	154	36.9
Five or more	263	63.1
Number of children under the age of five		
One	192	46.0
Two or more	225	54.0

### Household agricultural production and status of household food security

The mean (±SD) household agricultural land size of the households included in the study was 1.4 (±0.5) hectares. Four-in-five (80.6%) households were involved in livestock production. The assessment of household food security showed that only 16.8% of the families were food secured; whereas, the remaining 2.9, 50.1 and 30.2% had mild, moderate and severe food insecurity, respectively (Table 2).

**Table 2:** Household agricultural production and status of household food security in Gorche district, Southern Ethiopia, 2015

Variables and categories (*n* =417)	Frequency	Percentage
Proportion of households that:		
Grow Enset	410	98.3
Grow cereals	337	80.8
Own livestock	336	80.6
Grow fruits and vegetables	333	79.9
Grow cash crops	289	69.3
Grow legumes	202	48.4
Household food security status		
Food secured	70	16.8
Mildly food insecure	12	2.9
Moderately food insecure	209	50.1
Severely food insecure	126	30.2

### Level of dietary diversity

The mean (±SD) DDS calculated out of the standard seven food groups was 2.0 (±1.0) and only 10.6% (95% CI: 7.6-13.6) of the children had acceptable DD in the preceding day of the survey. About three-fourth (78%) of the children received solid, semi-solid, or soft foods of the minimum recommended frequencies, as defined in the WHO guideline [14]. Only 8.4% of the children had met the minimum acceptable diet standard – having the minimum DD and the minimum meal frequency during the previous day.

The majority (87.5%) of mothers reported that their children consumed complementary foods prepared from starchy staple foods including grains, roots and tubers in the reference period. Only a quarter (24.2%) received foods made from legumes and pulses. Vitamin A rich and other fruits and vegetables were consumed by 7.2 and 39.1% of the children, respectively. Regarding animal source foods, more than half (60.7%) received dairy and dairy products excluding breast milk (85.4%); nevertheless, eggs (11.0%) and meat (2.6%) were less frequently consumed.

### Factors associated with dietary diversity

A total of 20 variables were considered for the bivariate analysis. The following variables did not show significant association hence they were not considered for the multivariate analysis: child’s sex, mother’s and father’s occupation, household wealth index, sex of the head of the household, land size, the number of under five children in the household, birth order and women involvement in Income Generating Activities (IGA).

Fifteen variables met the inclusion criteria and hence included in the multivariate model. The variables were; mother’s age, child’s age, father’s and mother’s educational status, the size of the households, agro-ecology of the kebele, cultivation of fruits and vegetables, production of legumes and nuts, ownership of livestock, mother’s exposure to IYCF information on the mass media, during Postnatal Care (PNC) visit and through having discussion with Health Extension Workers (HEWs), mother’s participated in food cooking demonstration, husband involvement in IYCF and maternal knowledge on IYCF.

According to the final multivariate regression models, in children having literate fathers, the DD was increased by 0.26 as compared to their counterparts (*p* = 0.026). Whereas, children from households that grow fruits and vegetables and own livestock, the DDS was increased by 0.32 (*p* = 0.032) and 0.51 (*p* = 0.001) as compared to their counterparts and, respectively. As the age of the child increases by a month, the DD also increased by 0.04 (*p* = 0.001). Children born from mothers that received IYCF message during PNC, the DDS was increased by 0.21 (*p* = 0.037). Unit increase in maternal knowledge on IYCF was associated with 0.41 rise in DDS (*p = 0.001*). Other factors that were positively associated with the outcome variable were mothers participated in food demonstration, exposure to IYCF information on the mass media and husband involvement in IYCF (Table 3).

**Table 3:** Bivariate and multivariate linear regression analyses on the factors associated with dietary diversity among children 6–23 months of age in Gorche District, Southern Ethiopia, 2015

	Outputs of the analysis			
Variables and coding	Bivariate		Multivariate	
	β^a^	*P* value	β^a^	*P* value
Distal factors				
Mother age in years (18–44)	-0.02	0.038*	-0.013	0.301
Father education (0 = illiterate, 1 = literate)	0.38	0.001*	0.26	0.026*
Mother education (0 = illiterate, 1 = literate)	0.24	0.049*	-0.09	0.533
Size of the household (3–12)	-0.03	0.289	-0.02	0.696
Agro-ecology (0 = Dega, 1 = Woyinadega)	-0.29	0.005*	0.10	0.459
Grow fruits and vegetables (0 = No, 1 = Yes)	0.39	0.001*	0.32	0.032*
Grow legumes (0 = No, 1 = Yes)	0.25	0.009*	0.09	0.373
Own livestock (0 = No, 1 = Yes)	0.60	0.001*	0.51	0.001*
Received IYCF information on mass media in the last 1 month (0 = No, 1 = Yes)	0.38	0.001*	0.29	0.004*
Received IYCF information from HEWs in the last 1 month (0 = No, 1 = Yes)	0.26	0.016*	0.17	0.086
Participated in cooking demonstration in the last 6 months (0 = No, 1 = Yes)	0.25	0.009*	0.23	0.010*
Child age in months (6–23)	0.05	0.001*	0.04	0.001*
Received IYCF information during PNC visit (0 = No, 1 = Yes)	0.34	0.001*	0.21	0.037*
Proximate factors				
Husbands involvement in IYCF (0 = No, 1 = Yes)	0.23	0.018*	0.20	0.037*
Maternal knowledge (0–4)	0.44	0.001*	0.41	0.001*

^a^Unstandardized regression coefficient

* Significant at *P* value of 0.05

## Discussion

The study found only one-tenth of children received adequately diversified food while a higher proportion (78%) had optimal feeding frequency. Consequently, fraction of children that received the minimum acceptable diet – a combined indictor on the basis of feeding frequency and diversity – remained exceedingly low (< 10%). This signifies that most children failed to satisfy the minimum acceptable diet requirement largely due to sub-optimal DD. Other studies conducted in Ethiopia supports the current studies [5, 22]. According to DHS 2011, 48% of children had the acceptable meal frequency; nonetheless, proportions that met the minimum DD (4.3%) and acceptable diet (4.0%), were remarkably low [5]. The finding also shows, the district is lagging behind the national target that envisions to raise proportion of children with acceptable diet to 20% by 2015 [23].

The study concluded only 10.6% of children had the minimum DD and local complementary foods are mainly prepared from starchy staple foods. Furthermore, nutrient dense animal source foods like eggs and flesh foods are infrequently offered to children. Other studies from Ethiopia came up with parallel findings [17, 22, 24]. A study conducted in 2011 in a nearby Borcha district found that only 14% of children had met the minimum DD and only 3 and 2% of children respectively received flesh foods and eggs [17]. A summary of surveys conducted in Southern Ethiopia concluded that in six of the nine surveys included, proportions of children with optimal DD were less than 25% [25].

Husband’s educational status and their direct involvement in IYCF showed positive association with DD of children. Limited number of literatures empirically evaluates the role of husbands in IYCF practice. An unpublished study conducted in South Wollo Zone, North Ethiopia concluded that children living in households where husbands are directly involve in IYCF have a significant 13.7% rise in DDS. A qualitative study in Gambia reported that husbands have a major influence on infant feeding practice including the timing for initiating complementary foods [26].

In this study it was observed that production of fruits and vegetables and ownership of livestock is associated with significant rise in DDS. The finding indicates implementation of nutrition sensitive agriculture might be imperative for improvement diversity of children’s diet. A study conducted in South Africa supports the current study [27]. A study in Philippines concluded preschool children from households with gardens had higher DDS as compared with their counterparts [28].

Exposure to IYCF messages on the mass media showed significant association with DD. Other studies also availed consistent evidence. A secondary data analysis of Ethiopian DHS and another study from Northwest Ethiopia supports the study [22, 29]. A study in India also identified media exposure as a significant determinant of IYCF practice [30]. Media is usually considered as a credible source of health and nutrition information hence such messages are likely to be adopted.

Mothers who took part in cooking demonstrations were more likely to provide diversified complementary food to their children. A study from Peru concluded the same findings [31]. A mixed study conducted in Ethiopia witnessed the role of cooking demonstrations in enhancing the IYCF practice of rural women [32]. The finding is consistent to the understanding that cooking demonstration particularly benefits resource poor rural communities with limited formal education by providing hands-on learning experiences based on available recipes.

Unexpectedly, the study found no association between DD and, maternal education and household wealth index. This can be due to various reasons. Regarding maternal education, the study was conducted in predominately uneducated community where less than 20% of mothers were literates. Most of the literate mothers were also likely to have very brief exposure to formal education. Consequently, comparison of the DDS between literate and illiterate mothers may not be sensitive enough to show the real merits of maternal education. The null association with household wealth status can be due to the methodological weakness of wealth index. As wealth index is a relative, not absolute scale, it may not have a discriminatory power in setting, like the study area, where wealth is relatively homogenous.

The following limitation should be noted while interpreting the findings of the study. The DD was assessed on the basis of a single day recall hence it may not precisely show the usual dietary behavior of the community. Maternal knowledge and husbands’ involvement in IYCF were measured via non-standard scales hence misclassification errors cannot be excluded. Further, measurement of husbands’ involvement in IYCF can be liable to social desirability bias. As the study is cross-sectional causal inference may not be strong.

## Conclusions

Based on findings from this study, the majority (89.4%) of children had low dietary diversity and consumption of animal source foods, fruits and vegetables is relatively low. In the study district, the level of dietary diversity is extremely low. Exposure of mothers to nutrition education, promotion of husbands’ involvement in IYCF and implementation of nutrition sensitive agriculture can significantly improve dietary diversity of children.

## Additional file

Additional file 1: English version questionnaire used for the data collection. (DOCX 44 kb)

## Abbreviations

DD: Dietary dversity
DDS: Dietary diversity score
DHS: Demographic and health survey
FANTA: Food and nutrition technical assistance
HEWs: Health extension workers
IGA: Income generating activity
IRB: Institutional review board
IYCF: Infant and young child feeding
PNC: Postnatal care
SD: Standard deviation
SSA: Sub Saharan Africa
WHO: World Health Organization
