# Supplementary material for: Dietary diversity and associated factors among children 6-23 months of age in Gorche district, Southern Ethiopia: Cross-sectional study
Source: BMC Pediatr. 2017 Jan 9;17:6. doi: 10.1186/s12887-016-0764-x (PMC5223415; doi:10.1186/s12887-016-0764-x)
Supplement: Additional file 1: — English version questionnaire used for the data collection. (DOCX 44 kb) [file 12887_2016_764_MOESM1_ESM.docx]

## English Version Questionnaire

Background information of the household

Code of kebele­­­­­_________

Name of Respondent________________ Address______________

Date of visit. ____________________

| PART ONE: SOCIO-DEMOGRAPHIC CHARACTERSTICS | | | | | | |
| --- | --- | --- | --- | --- | --- | --- |
| S.No | Question | | | Choices | | Skip pattern |
| 101 | Sex of head of household | | | 1. Male 2. Female | |  |
| 102 | Age of mother | | | _________________years | |  |
| 103 | Marital Statuses of household | | | 1. Married/Living together 2. Not ever married 3. Divorced/Separated 4. Widowed | |  |
| 104 | Educational Status of father | | | 1. Illiterate 2. Read or Writes 3. Formal education _________grade 4. Higher education | |  |
| 105 | Educational Status of mother | | | 1. Illiterate 2. Read or Writes 3. Formal education ________grade 4. Higher education | |  |
| 106 | Occupation of father | | | 1. Employee 2. Merchant 3. Farmer 4. Petty trade 5. Other (specify) __________ | |  |
| 107 | Occupation of mother | | | 1. Employee 2. Merchant 3. Farmer/Housewife 4. Petty trade 5. Other (specify) __________ | |  |
| 108 | Household size (people who usually eat from the same pot) | | | In number___________ | |  |
| 109 | Number of children <5years in the household | | | In number __________ | |  |
| 110 | Religion | | | 1. Protestant 2. Orthodox 3. Muslim 4. Catholic 5. Other (specify)___________ | |  |
| 111 | Ethnicity | | | 1. Sidama 2. Amhara 3. Oromo 4. Other (specify) | |  |
| 112 | Agro-ecology | | | 1. Dega 2. Woyinadega 3. Kola 4. Other (specify)__________ | |  |
| PART TWO: SOCIO-ECONOMIC INFORMATION OF THE HOUSEHOLD | | | | | | |
| 201 | How is food obtained in family? (Probe for all responses) | | | 1. Farming (crops, animals) 2. Buying from market 3. Food aid/donation 4. Other (specify)_______ | |  |
| 202 | Do you own any cultivated land? | | | 1. Yes 2. No | | If No, skip to Q206 |
| 203 | If yes, how much is put for food production/agriculture? Give approximate average area in hectare | | | _________ in hectare | |  |
| 204 | Which of the following vegetables do you currently cultivate? (*Multiple answers possible)* | | | 1. Tomatoes 2. Potatoes 3. Onions 4. Cabbages 5. Corn 6. Carrots 7. Other (specify)__________ | |  |
| 205 | Which of the staple foods and other crops do you currently cultivate? (*Multiple answers possible)* | | | 1. Enset 2. Wheat 3. Teff 4. Barley 5. Rice 6. Pea 7. Bean 8. Maize 9. Other (specify)__________ | |  |
| 206 | Which of the following Cash crops do you currently cultivate? | | | 1. Chat 2. Coffee 3. Other (specify)__________ | |  |
| 207 | Currently your cultivated land is covered by what? | | | 1. Cash crops 2. Cereals 3. Enset 4. Other (specify)__________ | |  |
| 208 | Do you have animals (Domestic)? | | | 1. Yes 2. No | | If No, skip to Q212 |
| 209 | If yes, how many? | | | 1. Cows, Bulls, Oxen ______ 2. Horses, Donkeys, Mules__ 3. Goats ________ in number 4. Sheep ________ in number 5. Chicken ______ in number 6. Other (specify)__________ | |  |
| 210 | What is main current source of drinking water for members of your house hold? | | | 1. Piped water 2. Public Tap/Stand Pipe 3. Borehole 4. Protected well 5. Unprotected well 6. Protected Spring 7. Unprotected Spring 8. River/Ponds/Stream/Dam 9. Other (specify) _________ | |  |
| 211 | What is the main source of water used by your household for other purposes such as cooking and hand washing? | | | 1. Piped water 2. Public Tap/Stand Pipe 3. Borehole 4. Protected well 5. Unprotected well 6. Protected Spring 7. Unprotected Spring 8. River/Ponds/Stream/Dam 9. Other (specify)_________ | |  |
| 212 | What of the following does your household have? (*Multiple answers possible)* | | | 1. Electricity 2. Watch 3. Radio 4. Television 5. Mobile Telephone 6. Non Mobile Telephone 7. Chair 8. Table 9. Bed 10. Electric Mitad 11. Other (specify) _________ | |  |
| 213 | What kind of toilet facility do members of your household usually use? (Observe) | | | 1. Flush to piped sewer system 2. Flush to septic tank 3. Pit latrine with slab 4. Pit latrine without slab 5. Ventilated improved pit latrine (VIP). 6. No facility/bush/field 7. Other (specify)__________ | |  |
| 214 | What are the main materials of the floor of house? | | | 1. Earth/Sand 2. Wood planks 3. Palm/Bamboo 4. Ceramic Tiles 5. Cement 6. Other (specify)__________ | |  |
| 215 | What are the main materials of the roof of house? (*observe)* | | | 1. Thatch/straw 2. Leaf/Earth/ Mud/Cow dung 3. Wood planks, cardboard 4. Finished roof (iron, tin, finished wood, cement, ceramic) 5. Other (specify)__________ | |  |
| 216 | What are the main materials of the Walls of house? (*observe)* | | | 1. Simple wall with mud or local materials 2. Bamboo or stone with mud, plywood, cardboard 3. Finished walls; cement, brick, stone with cement, wood planks 4. No outside walls 5. Others (specify)_________ | |  |
| 217 | How many rooms are there in your house? | | | ______________ in number | |  |
| 218 | What type of fuel do you mainly use for cooking? (*Multiple answer possible)* | | | 1. Electricity 2. Wood 3. Kerosene 4. Animal dung 5. Charcoal 6. Shrubs/Grass 7. Agricultural crops 8. Other (specify)__________ | |  |
| PART THREE: WOMEN EMPOWERMENT AT HOUSEHOLD LEVEL | | | | | | |
| 301 | Do you have your own income generating activities? | | | 1. Yes  2. No | | If no, skip to Q303 |
| 302 | If yes, what? | | | 1. Farming 2. Rearing livestock 3. Trading 4. Other (specify)_________ | |  |
| 303 | Usually Who had final say in the following decisions? | | | | |  |
| 303.1 | How the money you earn will be used | | | 1. Mainly respondent/women/ 2. Mainly husband 3. Only respondent/women/ 4. Only husband 5. Both jointly | |  |
| 303.2 | Major household materials purchases | | | 1. Mainly respondent/women/ 2. Mainly husband 3. Only respondent/women/ 4. Only husband 5. Both jointly | |  |
| 303.3 | Household purchases for daily needs | | | 1. Mainly respondent/women/ 2. Mainly husband 3. Only respondent/women/ 4. Only husband 5. Both jointly | |  |
| 303.4 | Your own and your child health care | | | 1. Mainly respondent/women/ 2. Mainly husband 3. Only respondent/women/ 4. Only husband 5. Both jointly | |  |
| PART FOUR: MOTHER CHARACTERSTICS AND EXPOSURE TO NUTRITIO INTERVENTION. | | | | | | |
| 401 | Did you visit health facility for ANC during pregnancy? | | 1. Yes 2. No | | | If no, skip to Q406 |
| 402 | If yes, how many times you visited ANC during pregnancy? | | 1. 1 times 2. 2-3 times 3. 4-5 times 4. >5 times | | |  |
| 403 | Did you visit health facility for PNC? | | 1. Yes 2. No | | |  |
| 404 | If yes, how many times You visited? | | ________in number | | |  |
| 405 | Do you every attended any nutrition education session related to child feeding practices that given by health professionals in the last one month? | | 1. Yes 2. No | | |  |
| 406 | Do you every attended any nutrition education session related to child feeding practices that given by health extension workers in the last one month? | | 1. Yes 2. No | | |  |
| 407 | Do you every attended any nutrition education session related to child feeding practices that given by voluntary community health workers in the last one month? | | 1. Yes 2. No | | |  |
| 408 | Did you hear about infant and young child feeding practices by mass media like radio/television in the last one month? | | 1. Yes 2. No | | |  |
| 409 | Do you every participated any food cooking demonstration programme with in last six months? | | 1. Yes 2. No | | |  |
| PART FIVE: HUSBAND INVOLVEMENT ON INFANT AND YOUNG CHILD FEEDING PRACTICES (ASK MOTHER) | | | | | | |
| 501 | Does your husband discuss with you on the type of the food that provide for your child? | | 1. Yes 2. No | | |  |
| 502 | Does your husband give the money to you in order to buy and feed the foods (eggs, milk, meat…) to your child? | | 1. Yes 2. No | | |  |
| 503 | Does your husband buy and bring variety of foods (eggs, milk, meat…) to feed your child? | | 1. Yes 2. No | | |  |
| 504 | Does your husband follow up that your child consumes adequate amount of food? | | 1. Yes 2. No | | |  |
| PART SIX: MATERNAL KNOWLEDGE ON INFANT AND YOUNG CHILD FEEDING PRACTICES | | | | | | |
| 601 | Do you know for how many years feed breast milk to your child? *(check up to 2 years)* | | 1. Yes 2. No | | |  |
| 602 | Do you know that when you start complementary feeding for your child? *(refer Q705 below)* | | 1. Yes 2. No | | |  |
| 603 | Do you know what kind of foods to feed your child? *(refer part nine questions)* | | 1. Yes 2. No | | |  |
| 604 | Do you know that when you start family food for your child? | | 1. Yes  2. No | | |  |
| PART SEVEN: INFANT AND YOUNG CHILD FEEDING PRACTICES | | | | | | |
| 701 | Age of child in Month’s | | | __________ in month | |  |
| 702 | Sex of child | | | 1. Male 2. Female | |  |
| 703 | Birth order | | | 1. First 2. Second 3. Third 4. Fourth and above | |  |
| 704 | Are your child still breast feeding? | | | 1. Yes 2. No | |  |
| 705 | When did you start complementary food for your child? | | | ____________months (Yet to start complementary feeding) | |  |
| PART EIGHT: TO MEASURE HFIAS | | | | | | |
| 801 | In the past [4 weeks/30 days], did you worry that your household would not have enough food? | | | 1. Yes   2. No | | If No, skip to Q803 |
| 802 | If yes, How often did this happen in the past [4 weeks/30 days]? | | | 1. Rarely (1–2 times) 2. Sometimes (3–10 times) 3. Often (more than 10 times) | |  |
| 803 | In the past [4 weeks/30 days], were you or any household member not able to eat the kinds of foods you preferred because of a lack of resources? | | | 1. Yes  2. No | | If No, Skip to Q805 |
| 804 | How often did this happen in the past [4 weeks/30 days]? | | | 1. Rarely (1–2 times) 2. Sometimes (3–10 times) 3. Often (more than10 times) | |  |
| 805 | In the past [4 weeks/30 days], did you or any household member have to eat a limited variety of foods due to a lack of resources? | | | 1. Yes 2. No | | If no, Skip to the Q807 |
| 806 | If yes, How often did this happen in the past [4 weeks/30 days]? | | | 1. Rarely (1–2 times) 2. Sometimes (3–10 times) 3. Often (more than 10 times) | |  |
| 807 | In the past 4 weeks (30 days), did you or any household member have to eat some foods that you really did not want to eat because of a lack of resources to obtain other types of food? | | | 1. Yes 2. No | | If no, skip to the Q809 |
| 808 | If yes, How often did this happen in the past [4 weeks/30 days]? | | | 1. Rarely (1–2 times) 2. Sometimes (3–10 times) 3. Often (more than 10 times) | |  |
| 809 | In the past 4 weeks (30 days), did you or any household member have to eat a smaller meal than you felt you needed because there was not enough food? | | | 1. Yes 2. No | | If no, skip to the Q811 |
| 810 | If yes, How often did this happen in the past [4 weeks/30 days]? | | | 1. Rarely (1–2 times) 2. Sometimes (3–10 times 3. Often (more than 10) | |  |
| 811 | In the past 4 weeks (30 days), did you or any household member have to eat fewer meals in a day because there was not enough food? | | | 1. Yes 2. No | | If no, skip to the Q813 |
| 812 | If yes, How often did this happen in the past [4 weeks/30 days]? | | | 1. Rarely (1–2 times) 2. Sometimes (3–10 times 3. Often (more than10) | |  |
| 813 | In the past 4 weeks (30 days), was there ever no food to eat of any kind in your house because of lack of resources to get food? | | | 1. Yes 2. No | | If no, skip to the Q815 |
| 814 | If yes, How often did this happen in the past [4 weeks/30 days]? | | | 1. Rarely (1–2 times) 2. Sometimes (3–10 times 3. Often (>10) | |  |
| 815 | In the past 4 weeks (30 days), did you or any household member go to sleep at night hungry because there was not enough food? | | | 1. Yes 2. No | | If no, skip to the Q817 |
| 816 | If yes, How often did this happen in the past [4 weeks/30 days]? | | | 1. Rarely (1–2 times) 2. Sometimes (3–10 times 3. Often (more than 10) | |  |
| 817 | In the past 4 weeks (30 days), did you or any household member go a whole day and night without eating anything because there was not enough food? | | | 1. Yes 2. No | |  |
| 818 | If yes, How often did this happen in the past [4 weeks/30 days]? | | | 1. Rarely (1–2 times) 2. Sometimes (3–10 times 3. Often (more than 10) | |  |
| PART NINE**:** ASSESS DIETARY DIVERSITY AND FEEDING FREQUENCY AMONG CHILDREN AGED 6-23 MONTHS. DID THE CHILD EAT THE FOLLOWING FOODS THE PREVIOUS DAY AND NIGHT TIME WHETHER AT HOME OR OUTSIDE THE HOME? | | | | | | |
| S.No | FOOD GROUPS | EXAMPLES | | | CHOICES | |
| 901 | Grains, roots and tubers | Bread, chivada, rice, porridge, maize,  Wheat, Barley, enset | | | 1. Yes 2. No | |
| 902 | Legumes and nuts | Beans, peas, lentils, nuts, seeds or food made from these | | | 1. Yes 2. No | |
| 903 | Dairy products | milk, curds, cheese or other milk  products | | | 1. Yes 2. No | |
| 904 | Flesh foods | Pork, lamb, goat, wild game, Chicken, duck or other birds. Fresh or dried fish. Poultry, liver, kidney, heart, mutton and other organ meats or blood based food. | | | 1. Yes 2. No | |
| 905 | Eggs |  | | | 1. Yes 2. No | |
| 906 | Vitamin A rich fruits and vegetables | Ripe mangoes, dried amla, Pumpkin, carrots, squash, or sweet potatoes that are orange inside | | | 1. Yes 2. No | |
| 907 | Other fruits and  vegetables | Other fruits onions, tomatoes, cabbages, oranges, bananas, including wild fruits | | | 1. Yes 2. No | |
| 908 | How many times do you feed your child day and night time within 24-hours? | _____________ In number | | |  | |
